# Corneal subbasal nerve alterations and tear cytokine associations in ocular chronic graft-versus-host disease

**DOI:** 10.3389/fmed.2025.1662302

**Published:** 2025-09-10

**Authors:** Yinghan Zhao, Jiao Ma, Zhan Shen, Bohao Hu, Rongmei Peng, Shuwan Liu, Chendi Li, Jing Hong

**Affiliations:** ^1^Department of Ophthalmology, Peking University Third Hospital, Beijing, China; ^2^Beijing Key Laboratory of Restoration of Damaged Ocular Nerve, Peking University Third Hospital, Beijing, China

**Keywords:** ocular chronic graft-versus-host disease, tear cytokines, corneal subbasal nerve, cornea, hematopoietic stem cell transplant (HSCT)

## Abstract

**Objective:**

To determine the corneal subbasal nerve parameters associated with ocular chronic graft-versus-host disease (cGVHD) and their relationships with tear cytokine levels.

**Methods:**

Twenty-six ocular cGVHD patients, eight non-GVHD patients and 20 dry eye disease patients were recruited. The corneal subbasal nerve parameters were detected using *in vivo* confocal microscopy. The density and tortuosity scale of the corneal nerve were examined. Clinical characteristics, ocular surface parameters and tear cytokine levels, including NGF-β, G-CSF, GM-CSF, M-CSF, FGF1, FGF2, Fas-L, PDGF-CC, and CD137, were also analyzed.

**Results:**

Compared with DED patients, GVHD patients have significantly greater corneal subbasal nerve tortuosity and lower density; however, no differences were noted compared with non-GVHD patients. NGF-β, G-CSF, GM-CSF, M-CSF, FGF1, FGF2, Fas-L, and CD137 levels were evaluated in the tears of GVHD patients compared with those of DED patients. M-CSF, FGF1, and Fas-L were present at higher concentrations in GVHD patients than in non-GVHD patients. The PDGF-CC concentration was lower in the GVHD group compared with the non-GVHD group. M-CSF, GM-CSF, FGF1, FGF2, Fas-L, CD137, and PDGF-CC levels are associated with the density of the corneal subbasal nerve. M-CSF, GM-CSF, FGF1, FGF2, Fas-L, and CD137 levels correlated with the tortuosity of the subbasal nerve.

**Conclusion:**

In ocular cGVHD patients, the corneal subbasal nerve has a lower density and greater tortuosity but is similar to that in non-GVHD patients. IVCM findings of the corneal subbasal nerve are likely associated with tear cytokine changes.

**Clinical trial registration:**

Identifier ChiCTR2100051883.

## Introduction

Graft-versus-host disease (GVHD) remains a serious complication post hematopoietic stem cell transplantation (HSCT), with chronic GVHD (cGVHD) representing the most common long-term complication, affecting up to 70% of patients (1). Ocular cGVHD is among the most prevalent and debilitating complications, affecting 40%–60% of cGVHD patients. These manifestations range from dry eye disease (DED) and keratoconjunctivitis sicca (KCS) to meibomian gland dysfunction and, in severe cases, corneal perforation or even loss of vision (1).

Ocular cGVHD significantly impairs visual quality, also manifesting as discomfort or persistent eye pain of varying intensity. Previous studies have reported conflicting results on the relationship between symptoms and corneal subbasal nerve abnormalities. Some studies showing no significant difference in corneal subbasal nerve density between cGVHD patients and severe dry eye patients (2). Conversely, other investigations have documented notable reductions in corneal nerve density, increased tortuosity, and reduced reflectivity in ocular cGVHD individuals compared with normal participants and post-HSCT patients without ocular cGVHD. Additionally, reduced corneal sensitivity has been observed. However, the mechanisms underlying these structural nerve changes and their association with ocular pain remain incompletely understood (2–6).

The pathophysiology of ocular cGVHD involves immune-mediated damage to the lacrimal glands, conjunctiva, and corneal nerves, which results in reduced tear secretion, increased tear evaporation, and widespread ocular surface impairment. Among these, corneal nerve abnormalities, characterized by reduced density and increased tortuosity, have emerged as critical structural changes, closely linked to both inflammation and neurotrophic dysregulation. Notably, the prevalence of neurotrophic keratopathy in chronic oGVHD patients as been reported to reach up to 14% (7), suggesting that corneal nerve damage is common in patients with ocular cGVHD. Interestingly, even non-oGVHD post-HSCT patients exhibit subtle corneal nerve changes, suggesting that such alterations may result from post-HSCT ocular microenvironment remodeling rather than from GVHD itself (2). These findings highlight the need to better understand and monitor corneal nerve status in all post-HSCT recipients as a potential indicator of early diagnosis and a target for intervention.

Tear film plays a central role in modulating ocular immunity and nerve repair, as it contains a complex mixture of cytokines and neurotrophic agents. Previous studies have demonstrated significant alterations in the tear microenvironment in ocular GVHD, including IL-1β, IL-6, IL-8, IL-10, IL-17, and interferon-gamma (IFN-γ), along with CXCL9 and CXCL10 and matrix metalloproteinases (MMP-8 and MMP-9) (8–11). Notably, levels of vascular endothelial growth factor (VEGF) and extracellular DNA (eDNA) are also elevated, reflecting tissue damage and angiogenic processes. Conversely, reductions in epidermal growth factor (EGF) and its receptor (EGFR) have been observed, suggesting impaired epithelial repair (11). Collectively, these findings highlight a profoundly dysregulated inflammatory and repair environment in oGVHD.

Despite these insights, systematic research on how tear film factors influence corneal neuropathy in GVHD, particularly nerve tortuosity and nerve density, remains lacking. Beyond classic inflammatory cytokines, other mediators such as Fas-L, M-CSF, G-CSF, and PDGF-CC are known to regulate ocular surface immunity. Furthermore, NGF, PDGF-CC, FGF, M-CSF, and GM-CSF play significant roles in nerve regeneration and tissue repair processes. However, whether these cytokines and growth factors contribute to the pathogenesis of cGVHD remains unclear. Notably, corneal neurotization and vascularization have been shown to exert reciprocal inhibitory effects in neurotrophic keratopathy, underscoring the importance of considering both immune and angiogenic pathways in corneal pathology (12).

Building on this evidence, our study aimed to investigate whether tear film cytokines and neurotrophic factors contribute to corneal nerve alterations in ocular cGVHD.

## Materials and methods

A total of 34 patients attending the Ophthalmology Department after HSCT and 20 DED patients were enrolled in the study between June and December 2020. This prospective clinical study was registered, and was approved by the Ethics Committee, Informed consent was obtained from all the participants.

The diagnosis of ocular chronic GVHD was established by an experienced ophthalmologist based on a history of allogenic HSCT treatment and the presence of systemic and ocular symptoms according to the International Chronic oGVHD (ICCGVHD) consensus group diagnostic criteria (1).

Individuals who met the following criteria were included in the GVHD group: (1) diagnosed with ocular chronic GVHD according to the diagnostic criteria published by ICCGVHD; (2) first visited the ophthalmology clinic after HSCT; and (3) without ocular immunosuppressant treatment. Patients were excluded on the basis of the following criteria: (1) eye allergies, infections, glaucoma, retinopathy, or other systemic immune system diseases that could affect the ocular surface; (2) lack of complete medical records or clinical records; (3) ocular immunosuppressant treatment; and (4) diagnosis of diabetic keratopathy or neurotrophic keratopathy. DED patients were diagnosed according to the Tear Film & Ocular Surface Society Dry Eye Workshop II (TFOS DEWS II) criteria. Inclusion criteria included Ocular Surface Disease Index (OSDI) score ≥ 13, tear break-up time (TBUT) ≤ 5 s, and/or corneal fluorescein staining (CFS) ≥ 1 point, with Schirmer I test values > 5 mm.

The main clinical symptoms were evaluated using the ocular surface disease index (OSDI) questionnaire for each patient. All ocular clinical examinations, including noncontact intraocular pressure (IOP, mmHg), best-corrected visual acuity (BCVA, LogMAR), CFS, TBUT (s), tear meniscus (TM, mm) and Schirmer’s test (mm), were performed at the patients’ first visit. CSF was measured in five areas of the cornea, including the center, upper, lower, left, and right areas. Each area was scored on a scale of 0–3. A score of 0 was assigned for no staining; one point for fewer than 15 punctate staining spots; two points for 15–30 spots; and three points for more than 30 spots or confluent staining areas.

We prospectively collected and investigated tear cytokine samples from each group. Tear samples were obtained from both eyes in post-HSCT patients, and from one affected eye in patients with DED. First, 30 μL of sterile normal saline solution was added to the conjunctival sac, and 15 μL of tear fluid was collected using a capillary tear collector. For each examined eye, tear fluid was collected three times and mixed into one centrifuge tube to analyze (8). All tear samples were stored in a – 80 °C freezer until further examination. Cytokine concentrations were detected using microsphere-based immunoassay analysis (Seinda Biomedical Corporation, Guangdong, China) (13, 14).

IVCM was used to examine the central cornea in all patients. Three representative high-quality images of subbasal nerves in the central cornea of each patient were randomly selected for image analysis, observed, and analyzed using ImageJ software. Identifying information was masked during the blind analysis process.

Subbasal nerve density was defined as the total length of nerve fibers traced per image area, measured in mm/mm (2). Neuron J, a plug-in program of ImageJ provided in the public domain by Erik Meijering, was used as the analysis tool for measuring subbasal nerve density. The tortuosity of the subbasal nerves was graded from 0 to 4, according to previous research (15). Three different images were evaluated for each individual, and the mean of the three measurements was used for analysis.

Statistical analyses were performed using IBM SPSS Statistics version 26. The statistical significance level was set at 5%. The Shapiro–Wilk test was used to check the normality of the data. Crosstabs and chi-square tests were used to analyze categorical variables. The nonparametric Kruskal–Wallis test was used to compare continuous variables among the three groups if the normality assumption was not met. Student’s *t*-test for two independent samples was used if the data were normally distributed. Spearman’s rank correlation tests were used to assess the correlations between the levels of cytokines and subbasal nerve characteristics and clinical features.

## Results

A total of 26 patients with ocular cGVHD (41 eyes), eight non-GVHD patients (14 eyes), and 20 patients with DED (20 eyes) were included in the study. No significant difference in age was noted between the two groups (*p* = 0.062). The primary hematological diseases found in the non-GVHD group were myelodysplastic syndrome (MDS, *n* = 3), chronic myelogenous leukemia (CML, *n* = 2), acute lymphoblastic leukemia (ALL, *n* = 2), and acute myeloid leukemia (AML, *n* = 2). The primary hematological diseases in the GVHD group were AML (*n* = 10), ALL (*n* = 6), MDS (*n* = 6), aplastic anemia (AA, *n* = 2), lymphadenoma (*n* = 1), and plasma cell leukemia (PCL, *n* = 1). No significant differences were noted in the HLA-matched degree of HSCT therapy (*p* = 0.383). Other organ involvement in cGVHD is shown in Table 1. The time interval from HSCT to ophthalmologic assessment was significantly longer in the GVHD group (737.67 days) compared to the non-GVHD group (285.13 days), with a *p*-value of 0.003, indicating a statistically significant difference.

**Table 1:** Basic characteristics of participants in the non-GVHD group and the GVHD group.

Characteristics	DED group (*n* = 20)	Non-GVHD group (*n* = 8)	GVHD group (*n* = 26)
Sex (%)			
Male	14 (70%)	6 (75%)	11 (42.3%)
Female	6 (30%)	2 (25%)	15 (57.7%)
HLA matched degree (%)			
HLA identical	/	6 (75%)	11 (42.3%)
HLA non-identical	/	2 (25%)	15 (57.7%)
Other organ cGVHD (%)	/		
Skin	/	6 (75%)	18 (69.2%)
Oral	/	4 (50%)	21 (80.8%)
Intestinal	/	1 (12.5%)	5 (19.2%)
Lung	/	1 (12.5%)	5 (19.2%)
Liver	/	2 (25%)	7 (26.9%)

The data are presented as percentages. Crosstabs and chi-square tests were used.

The ocular clinical features are presented in Table 2. BCVA was significantly worse in the GVHD group (0.22 ± 0.17) compared to the DED group (0.05 ± 0.10, *p* < 0.001), but not significantly different from the non-GVHD group (0.34 ± 0.08, *p* = 0.624). No significant differences in intraocular pressure were observed among the three groups. OSDI score did not differ significantly between GVHD and DED groups (*p* = 0.362) or between GVHD and non-GVHD groups (*p* = 0.138).

**Table 2:** Clinical features of participants in the DED, non-GVHD, and GVHD groups.

Clinical parameters	DED (*n* = 20)	Non-GVHD	GVHD	*p*-value	
				GVHD vs. DED	GVHD vs. Non-GVHD
Age	39.40 ± 13.47	35.00 ± 16.00 (*n* = 8)	34.50 ± 14.00 (*n* = 26)		0.062
Time interval from HSCT (day)	/	285.13 (*n* = 8)	737.67 (*n* = 26)	/	0.003**
BCVA (LogMAR)	0.05 ± 0.10	0.34 ± 0.08 (*n* = 14)	0.22 ± 0.17 (*n* = 41)	<0.001***	0.624
Eye pressure (mm Hg)	14.70 ± 2.22	14.44 ± 3.32 (*n* = 14)	13.30 ± 1.58 (*n* = 41)	0.448	0.258
OSDI score	37.20 ± 19.60	27.08 ± 15.70 (*n* = 14)	34.73 ± 20.16 (*n* = 41)	0.362	0.138
Corneal fluorescein staining (CFS)	0.10 ± 0.31	0.00 ± 1.00 (*n* = 14)	6.00 ± 3.50 (*n* = 41)	<0.001***	<0.001***
Tear break-up time (BUT, s)	3.44 ± 1.36	3.00 ± 1.00 (*n* = 14)	2.00 ± 0.63 (*n* = 41)	0.013*	<0.001***
Tear meniscus (mm)	0.06 ± 0.03	0.20 ± 0.05 (*n* = 14)	0.10 ± 0.03 (*n* = 41)	<0.001***	<0.001***
Schirmer’s test (mm)	13.22 ± 7.81	5.47 ± 3.44 (*n* = 14)	1.00 ± 0.52 (y = 41)	<0.001***	<0.001**

The data are presented as the means ± SDs. The Mann–Whitney U test or Student’s *t-*test was used. *p*-values less than 0.05 were considered significant. *p < 0.05, **p < 0.01, ***p < 0.001.

All ocular surface clinical examinations revealed significant differences in the DED, non-GVHD, and GVHD groups. CFS was significantly greater in the GVHD group than in both of the non-GVHD group (6.00 ± 3.50 vs. 0.00 ± 1.00, *p* < 0.001) and the DED group (vs. 0.10 ± 0.31, *p* < 0.001). Similarly, the TBUT was much shorter in the GVHD group (2.00 ± 0.63) than in other two groups (non-GVHD 3.00 ± 1.00, *p* < 0.001, DED 3.44 ± 1.36, *p* = 0.013). Additionally, TM differed across groups, being lowest in DED (0.06 ± 0.03 mm), intermediate in GVHD (0.10 ± 0.03 mm), and highest in non-GVHD controls (0.20 ± 0.05 mm). Pairwise comparisons were significant (both *p* < 0.001). Schirmer’s test values were markedly reduced in GVHD patients (1.00 ± 0.52) compared to the DED (13.22 ± 7.81) and non-GVHD (5.47 ± 3.44) groups, with both comparisons reaching statistical significance (*p* < 0.001).

**Figure 1:**
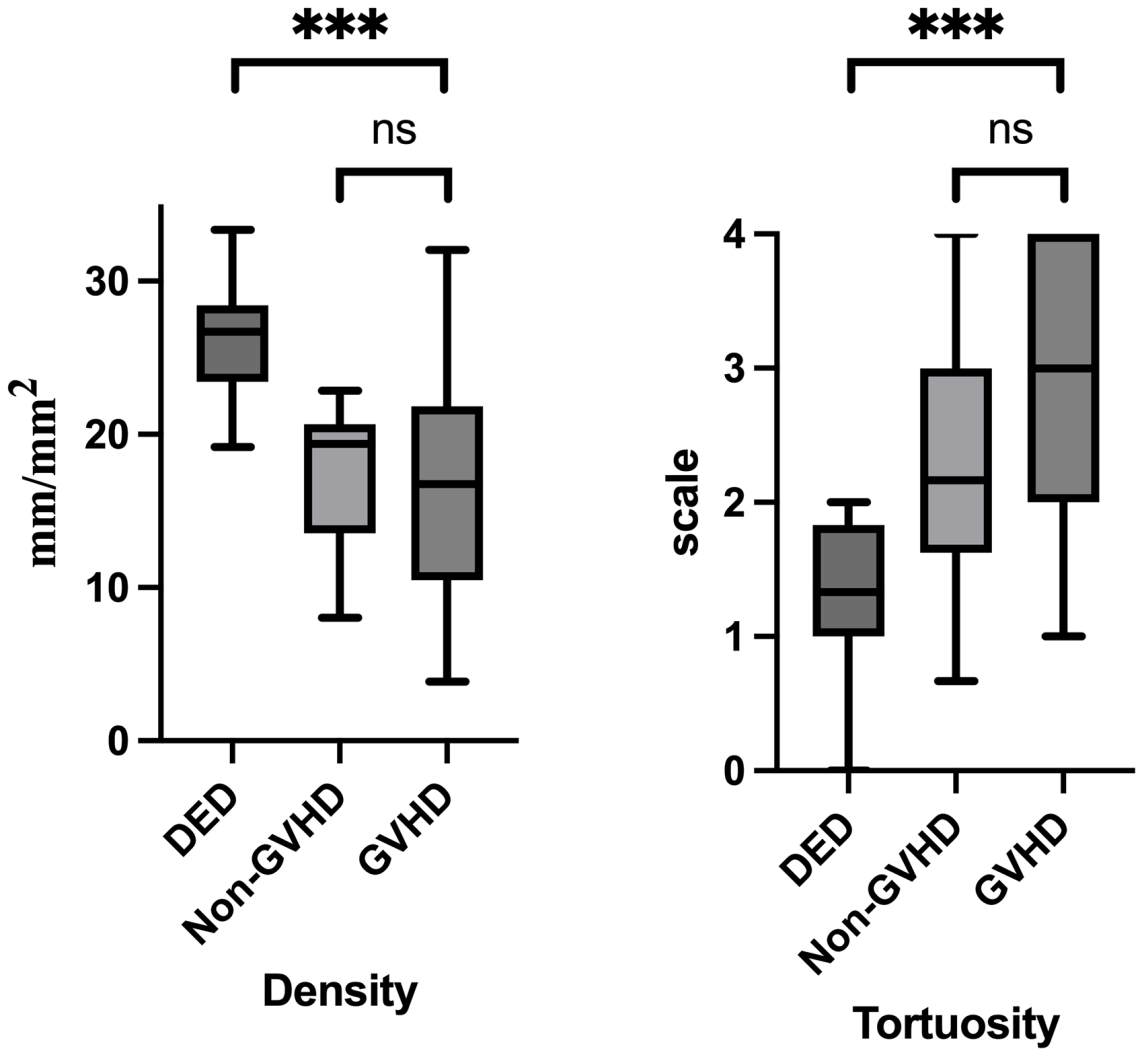
Comparison of corneal subbasal nerve density and tortuosity among patients with dry eye disease (DED), post-HSCT patients without graft-versus-host disease (non-GVHD), and post-HSCT patients with ocular GVHD. Box plots illustrate median, interquartile range, and overall range. The data are presented as the means ± SDs. The Mann–Whitney U test or Student’s *t*-test was used. *p-*values less than 0.05 were considered significant. *p < 0.05, **p < 0.01, ***p < 0.001.

Table 3 shows the results obtained from IVCM images of corneal subbasal nerve density and nerve tortuosity characteristics. The subbasal nerve density was 26.36 ± 3.82 mm/mm^2^ in the DED group, 17.34 ± 4.68 mm/mm^2^ in the non-GVHD group, and 16.57 ± 7.76 mm/mm^2^ in the GVHD group. Subbasal nerve density was significantly reduced in the GVHD group compared to DED group (*p* < 0.001), and in the non-GVHD group compared to DED group (*p* < 0.001). However, no significant differences were observed between the non-GVHD group and GVHD group (*p* = 0.816). In the GVHD group, subbasal nerve tortuosity was significantly greater than that in the DED group (2.93 ± 1.031 vs. 1.32 ± 0.50, *p* < 0.001) but was not significantly different from that in the non-GVHD group (2.25 ± 0.85, *p* = 0.078), shown in Figure 1. No significant correlation was observed between corneal nerve density and post-HSCT duration (*ρ* = 0.036, *p* = 0.84), and between nerve tortuosity and post-HSCT duration (*ρ* = 0.069, *p* = 0.71).

**Table 3:** Corneal subbasal nerve features by IVCM in the three groups.

Corneal subbasal nerve features	DED (*n* = 20)	Non-GVHD (*n* = 14)	GVHD (*n* = 41)	*p*-value		
				GVHD vs. DED	GVHD vs. Non-GVHD	Non-GVHD vs. DED
Density (mm/mm^2^)	26.36 ± 3.82	17.34 ± 4.68	16.57 ± 7.76	<0.001***	0.816	<0.001***
Tortuosity scale (0–4)	1.32 ± 0.50	2.25 ± 0.85	2.93 ± 1.031	<0.001***	0.078	<0.001***

The data are presented as the means ± SDs. The Mann–Whitney U test or Student’s *t*-test was used. *p*-values less than 0.05 were considered significant. *p < 0.05, **p < 0.01, ***p < 0.001.

**Figure 2:**
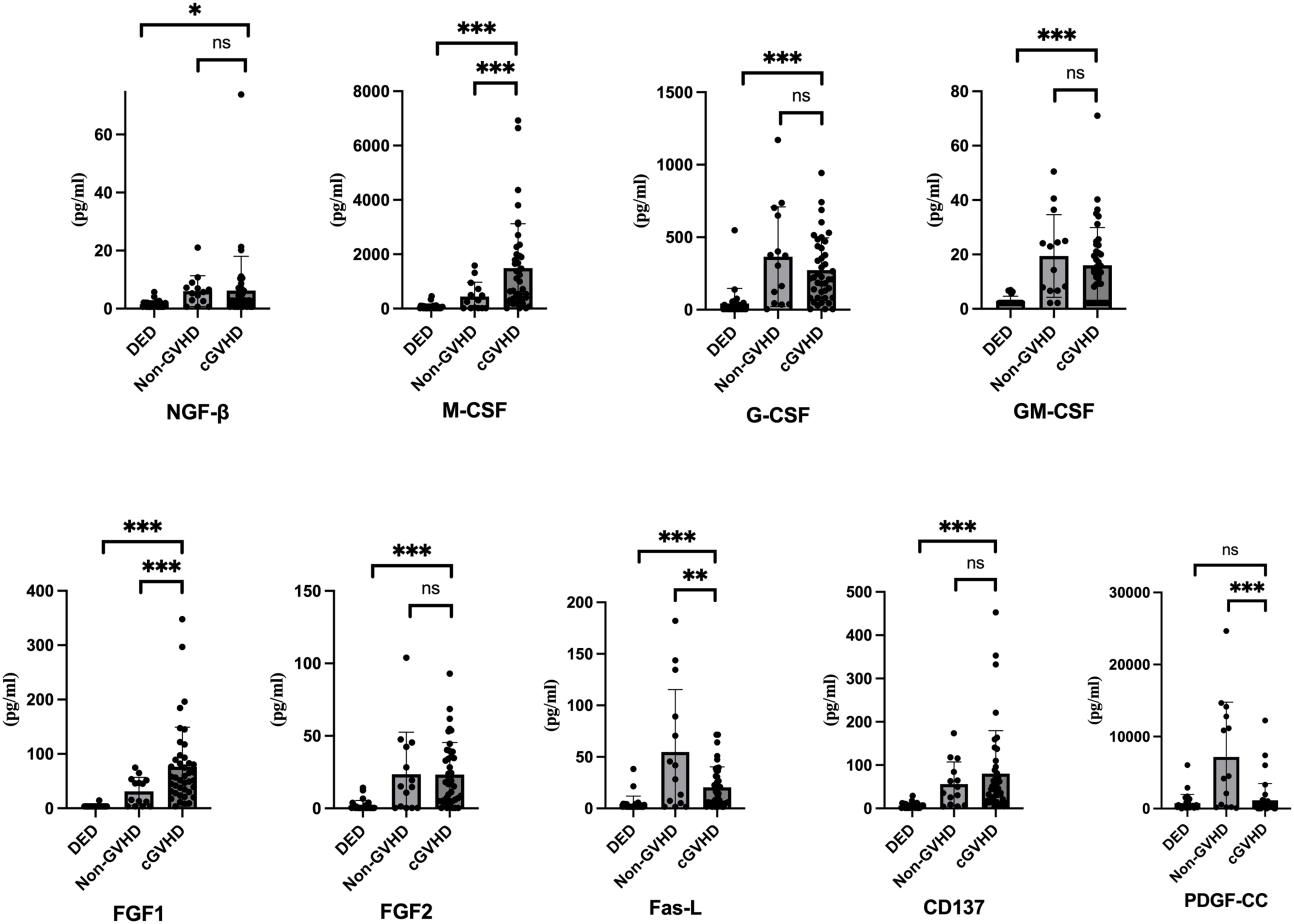
Correlation between tear cytokine concentrations and corneal nerve parameters among patients with dry eye disease (DED), post-HSCT patients without graft-versus-host disease (non-GVHD), and post-HSCT patients with ocular GVHD. The data are presented as the means ± SDs and were analyzed via the Mann–Whitney U test. *p*-values less than 0.05 were considered significant. *p < 0.05, **p < 0.01, ***p < 0.001.

The levels of various cytokines in the tear fluid of the three groups are presented in Table 4 and Figure 2. M-CSF levels were markedly elevated in the GVHD group (1490.20 ± 1635.54) compared with both the DED (68.23 ± 100.38) and non-GVHD groups (445.18 ± 521.69), with *p-*values less than 0.001 in both comparisons. Moreover, the GM-CSF levels were greater in the GVHD group than in the DED group (16.00 ± 13.89 vs. 2.92 ± 1.68, *p* < 0.001); however, the difference was not statistically significant between the GVHD and non-GVHD groups (19.43 ± 15.18, *p* = 0.465). Similarly, G-CSF levels were increased in the GVHD group compared with DED group (273.53 ± 222.18 vs. 42.85 ± 104.12, *p* < 0.001), and no significant difference was noted between the GVHD and non-GVHD groups (366.13 ± 343.70, *p* = 0.395). Fas-L levels were lower in the GVHD group than in the non-GVHD group (20.52 ± 19.82 vs. 54.74 ± 60.64, *p* = 0.002) but increased compared to the DED group (4.38 ± 7.66, *p* < 0.001). CD137 expression was significantly greater in both the GVHD (80.09 ± 99.42) and non-GVHD (56.08 ± 51.21) groups than in the DED (6.35 ± 6.45) group, but no significant difference was noted between the GVHD and non-GVHD groups.

**Table 4:** Levels of different cytokines in the tear fluid of the three groups.

Cytokines (pg/mL)	DED group (*n* = 20)	Non-GVHD group (*n* = 14)	GVHD group (*n* = 41)	*p*-value	
				GVHD vs. DED	GVHD vs. Non-GVHD
NGF-β	1.52 ± 1.18	6.02 ± 5.33	6.23 ± 11.79	0.015*	0.930
M-CSF	68.23 ± 100.38	445.18 ± 521.69	1490.20 ± 1635.54	<0.001***	<0.001***
G-CSF	42.85 ± 104.12	366.13 ± 343.70	273.53 ± 222.18	<0.001***	0.359
GM-CSF	2.92 ± 1.68	19.43 ± 15.18	16.00 ± 13.89	<0.001***	0.465
FGF1	3.79 ± 2.12	31.19 ± 25.79	76.55 ± 72.70	<0.001***	0.001**
FGF2	1.89 ± 3.56	23.61 ± 28.91	23.30 ± 22.13	<0.001***	0.967
Fas-L	4.38 ± 7.66	54.74 ± 60.64	20.52 ± 19.82	<0.001***	0.002**
CD137	6.35 ± 6.45	56.08 ± 51.21	80.09 ± 99.42	<0.001***	0.2521
PDGF-CC	763.46 ± 1213.23	7166.19 ± 7612.67	1170.15 ± 2299.68	0.343	<0.001***

The data are presented as the means ± SDs and were analyzed via the Mann–Whitney U test. *p-*values less than 0.05 were considered significant. *p < 0.05, **p < 0.01, ***p < 0.001.

FGF1 levels were significantly greater in the GVHD group (76.55 ± 72.70) than in the DED (3.79 ± 2.12) and non-GVHD (31.19 ± 25.79) groups, with *p-*values of <0.001 and 0.001, respectively. On the other hand, NGF-β levels were not significantly different between the GVHD and non-GVHD groups (*p* = 0.930). FGF2 levels were greater in the GVHD group than in the DED group (23.30 ± 22.13 vs. 1.89 ± 3.56, *p* < 0.001).

Table 5 presents the correlation (*r*) and associated *p-*values between specific cytokines and the density and tortuosity of subbasal nerves. For density, significant negative correlations were observed with M-CSF (*r* = −0.316, *p* = 0.006), GM-CSF (*r* = −0.336, *p* = 0.003), FGF1 (*r* = −0.346, *p* = 0.002), FGF2 (*r* = −0.410, *p* = 0.001), Fas-L (*r* = −0.244, *p* = 0.035), CD137 (*r* = −0.280, *p* = 0.015), and PDGF-CC (*r* = −0.245, *p* = 0.034). For tortuosity, significant positive correlations were identified with M-CSF (*r* = 0.367, *p* = 0.001), G-CSF (*r* = 0.280, *p* = 0.015), GM-CSF (*r* = 0.242, *p* = 0.036), FGF1 (*r* = 0.435, *p* < 0.001), FGF2 (*r* = 0.252, *p* = 0.029), and CD137 (*r* = 0.311, *p* = 0.007). Notably, PDGF-CC was significantly correlated with density (*p* = 0.034) but was not significantly correlated with tortuosity (*p* = 0.994). These findings suggest distinct relationships between cytokine levels and subbasal nerve parameters.

**Table 5:** Correlation between the concentrations of tear cytokines and corneal subbasal nerve features in GVHD patients.

Cytokines	Density of subbasal nerve		Subbasal nerve tortuosity	
	*r*	*p*-value	*r*	*p*-value
NGF-β	−0.156	0.181	0.083	0.478
M-CSF	−0.316	0.006**	0.367	0.001**
G-CSF	−0.135	0.247	0.280	0.015*
GM-CSF	−0.336	0.003**	0.242	0.036*
FGF1	−0.346	0.002**	0.435	<0.001***
FGF2	−0.410	0.001**	0.252	0.029*
Fas-L	−0.244	0.035*	0.253	0.029*
CD137	−0.280	0.015*	0.311	0.007**
PDGF-CC	−0.245	0.034*	0.001	0.994

*p-*values less than 0.05 were considered significant. *p < 0.05, **p < 0.01, ***p < 0.001.

## Discussion

The findings of this research provide new insights into the pathogenesis of chronic ocular cGVHD, by linking corneal subbasal nerve alterations to tear cytokine profile. Our study analyzed the morphology and density of corneal subbasal nerves under IVCM and measured cytokine levels in tear fluid, and analyzed the correlation between tear cytokine levels and corneal nerve injury repair.

The earliest study on corneal nerve alterations in GVHD patients reported by Kheirkhah et al. (2) revealed no significant differences in subbasal corneal nerve density between GVHD patients and non-GVHD patients. Similar findings were subsequently reported by He et al. (6) However, Tepelus et al. (5) reported that, compared with patients with patients with DED, GVHD patients presented with lower corneal nerve density, reduced reflectivity, and the presence of bead-like structures in the nerves. Whereas, Dikmetas et al. (4) demonstrated that GVHD patients had lower subbasal corneal nerve plexus density compared to those with severe dry eyes. Our study expands on these findings by comparing cGVHD patients not only to DED patients but also to non-GVHD after HSCT. These observations revealed that morphological changes in the corneal subbasal nerves are already present in post-HSCT patients, regardless of an ocular cGVHD diagnosis. These observations suggest that alterations in the subbasal corneal nerve begin to develop following the transplantation itself.

Tear cytokine levels serve as a marker to assess the ocular surface microenvironment and the degree of inflammation. Previous studies have suggested that changes in tear cytokines represent potential biomarkers for the early diagnosis of cGVHD (8). In our study, we first discovered that the levels of NGF, G-CSF, GM-CSF, M-CSF, FGF1, FGF2, Fas-L, and CD137 were significantly elevated post-HSCT, whereas M-CSF, FGF1, Fas-L, and PDGF-CC levels were able to distinguish between patients with and without GVHD after HSCT.

Several key molecular factors were found to be altered in the tear fluid, reflecting their potential roles in corneal nerve injury and repair. FGF1 and FGF2, known for their contributions to neural circuit development, axonal growth, and nerve regeneration (16, 17), showed significant differences post-HSCT and correlated with corneal nerve tortuosity and density in our study. This suggests FGFs may regulate corneal nerve injury processes after HSCT. The FGFR2 signaling pathway, which is critical for meibomian gland homeostasis and regeneration, may influence tear film composition (18, 19). Therefore, the relationship between meibomian gland abnormalities and FGF in GVHD patients remains to be further elucidated in future studies. Conversely, the lower levels of PDGF-CC in cGVHD patients compared to the non-GVHD group suggest a potential deficiency in a protective or reparative pathway.

CSFs, including GM-CSF and M-CSF, are traditionally considered regulators of hematopoiesis and macrophage/granulocyte activation (20–23). GM-CSF signaling is known to influence nervous system function (21), and plant-derived rhGM-CSF increases the corneal wound healing rate *in vivo* (24), but further research is needed to clarify its role in corneal nerve repair. Additionally, in a murine dry eye model, expression of GM-CSF was found to be significantly upregulated in both the conjunctiva and cornea, indicating that chronic ocular surface inflammation alone could affect tear cytokine levels (25). This finding may underscore that tear cytokine alterations may partly reflect not only corneal nerve changes but also ocular tissue inflammation. Recent experimental data also demonstrated that administration of IL-1β and IL-34 promotes corneal nerve regeneration in diabetic neurotrophic keratopathy, further suggesting that cytokines may act as direct modulators of neural repair (26).

CD137, a TNFR family member crucial for immune modulation (27), and Fas-L, a TNF superfamily glycoprotein involved in inflammatory response and neurodegeneration, were found to be increased in GVHD patients’ tears, suggesting their contribution to immune-mediated nerve damage. PDGF-CC, a neuroprotective factor that involved in skin wound repair (28, 29), was significantly higher in non-GVHD group, which reflect its potential role in neuroprotection. Our study found that PDGF-CC, observed to be higher in the non-GVHD group, suggests its potential as a therapeutic agent to preserve nerve integrity and promote tissue repair. NGF is a neurotrophic factor which is essential for nerve (30). It was elevated in the GVHD group compared to DED, consistent with its role in injury repair. These findings collectively highlight a complex interplay of growth factors and immune mediators in the ocular surface microenvironment following HSCT, influencing corneal nerve health and repair processes. The notable prevalence of neurotrophic keratopathy in cGVHD patients further emphasizes the severity of corneal nerve involvement in this condition. The significant incidence of neurotrophic keratopathy in patients with cGVHD further emphasizes that the severity of corneal nerve involvement in this disease requires close attention using IVCM.

Our study found those with GVHD showed significant differences in CFS BUT, tear meniscus, and Schirmer test results compared to DED patients and non-GVHD patients. However, no significant difference was observed in OSDI scores, suggesting a potential symptom–sign dissociation in GVHD. Previous studies have reported a 14% prevalence of neurotrophic keratopathy in GVHD patients (7), and reduced corneal sensitivity in this GVHD patients may be one of the contributing factors to our observed phenomenon.

Our study has significant clinical value for the diagnosis, prognosis, and potentially therapeutic management of ocular cGVHD. The observed alterations in corneal subbasal nerves, as objectively detected by IVCM, suggest its potential as a non-invasive tool for early disease detection and monitoring of disease progression. Furthermore, the identified tear cytokine profiles, particularly those distinguishing between GVHD and non-GVHD patients post-HSCT (M-CSF, FGF1, Fas-L, and PDGF-CC), could serve as objective biomarkers for disease activity. Moreover, from a therapeutic perspective, our study provides new understanding of these molecular factors, which opens new avenues for targeted interventions.

This study is limited by the lack of longitudinal observation of progressive changes in corneal nerve morphology in relation to disease severity. In future studies, standardized ocular pain should be systematically assessed, and it will be interesting to investigate longitudinal follow-up of non-GVHD patients to monitor disease progression more accurately, by focusing on all involved tissues, such as conjunctiva, meibomian glands, and lacrimal glands. Because the IVCM scans capture only a small region of the cornea, it is possible that variations in subbasal nerve distribution were not fully captured. Future studies incorporating wide-area mapping imaging could provide a more comprehensive evaluation of corneal nerve morphology and improve the accuracy of analyses. Finally, further studies with larger sample sizes are needed to evaluate the changes in corneal nerve across different severity levels. Additionally, in future investigations, direct corneal vascularization should be assessed, to better capture the complexity of neurovascular crosstalk in ocular GVHD, such as VEGF, PEDF and other angiogenic factors should be considered. In order to gain more detailed morphological and functional alternations of GVHD corneal nerve, future work should incorporate animal models to observe corneal nerve injury and regeneration, and provide complementary insights into the underlying mechanisms.

## Conclusion

In conclusion, this study provides evidence of significant corneal subbasal nerve damage in patients post-HSCT, particularly in those with ocular cGVHD revealed by IVCM. These structural changes, including reduced nerve density and increased tortuosity, suggest that corneal nerve alterations begin early after HSCT and could serve as potential early markers. Concurrently, the study identified elevated levels of neurotrophic factors (NGF, FGF1, FGF2), colony-stimulating factors (G-CSF, GM-CSF, M-CSF), and immune mediators (CD137, Fas-L) post-HSCT. Additionally, M-CSF, FGF1, Fas-L, and PDGF-CC levels can differentiate between patients with and without GVHD after HSCT. The correlation between FGF1, FGF2, GM-CSF, M-CSF and corneal nerve parameters reflects the complex relationship between ocular surface microenvironment and corneal nerve integrity of cGVHD. These findings suggest that IVCM and tear cytokine analysis may serve as valuable tools for early diagnosis of ocular cGVHD, monitoring disease progression, and guiding the development of future targeted therapies, ultimately improving corneal health and significantly improving the long-term quality of life of patients.

## Data Availability

The original contributions presented in the study are included in the article/supplementary material, further inquiries can be directed to the corresponding author.

## References

[1] Soleimani M Mahdavi Sharif P Cheraqpour K Koganti R Masoumi A Baharnoori SM. Ocular graft-versus-host disease (oGVHD): from A to Z. Surv Ophthalmol. (2023) 68:697–712. doi: 10.1016/j.survophthal.2023.02.006, PMID: 36870423 PMC10293080

[2] Kheirkhah A Qazi Y Arnoldner MA Suri K Dana R. In vivo confocal microscopy in dry eye disease associated with chronic graft-versus-host disease. Invest Ophthalmol Vis Sci. (2016) 57:4686–91. doi: 10.1167/iovs.16-20013, PMID: 27607414

[3] Setu MAK Schmidt S Musial G Stern ME Steven P. Segmentation and evaluation of corneal nerves and dendritic cells from in vivo confocal microscopy images using deep learning. Transl Vis Sci Technol. (2022) 11:24. doi: 10.1167/tvst.11.6.24, PMID: 35762938 PMC9251793

[4] Dikmetas O Kocabeyoglu S Mocan MC. The association between Meibomian gland atrophy and corneal subbasal nerve loss in patients with chronic ocular graft-versus-host disease. Curr Eye Res. (2021) 46:796–801. doi: 10.1080/02713683.2020.1846754, PMID: 33427504

[5] Tepelus TC Chiu GB Maram J Huang J Chopra V Sadda SVR. Corneal features in ocular graft-versus-host disease by in vivo confocal microscopy. Graefes Arch Clin Exp Ophthalmol. (2017) 255:2389–97. doi: 10.1007/s00417-017-3759-x, PMID: 28875340

[6] He J Ogawa Y Mukai S Saijo-Ban Y Kamoi M Uchino M. In vivo confocal microscopy evaluation of ocular surface with graft-versus-host disease-related dry eye disease. Sci Rep. (2017) 7:10720. doi: 10.1038/s41598-017-10237-w, PMID: 28878217 PMC5587759

[7] Singh RB Yuksel E Sinha S Wang S Taketani Y Luznik Z. Prevalence of neurotrophic keratopathy in patients with chronic ocular graft-versus-host disease. Ocul Surf. (2022) 26:13–8. doi: 10.1016/j.jtos.2022.07.001, PMID: 35843560

[8] Hu B Qiu Y Hong J. Tear cytokine levels in the diagnosis and severity assessment of ocular chronic graft-versus-host disease (GVHD). Ocul Surf. (2020) 18:298–304. doi: 10.1016/j.jtos.2019.12.005, PMID: 31954196

[9] Shen Z Ma J Peng R Hu B Zhao Y Liu S. Biomarkers in ocular graft-versus-host disease: implications for the involvement of B cells. Transplant Cell Ther. (2022) 28:749.e1–7. doi: 10.1016/j.jtct.2022.07.023, PMID: 35914728

[10] Qiu Y Hu B Peng RM Huang JF Hong J. Tear cytokines as biomarkers for acute ocular graft-versus-host disease. Cornea. (2022) 41:1405–11. doi: 10.1097/ICO.0000000000002959, PMID: 35184125

[11] Liu S Peng R Ma J Shen Z Hu B Zhao Y. Assessment of corneal epithelial changes and related factors in ocular chronic graft-versus-host disease (GVHD) by in vivo confocal microscopy. Ocul Immunol Inflamm. (2024) 32:454–62. doi: 10.1080/09273948.2023.2173240, PMID: 36758227

[12] Ferrari G Hajrasouliha AR Sadrai Z Ueno H Chauhan SK Dana R. Nerves and neovessels inhibit each other in the cornea. Invest Ophthalmol Vis Sci. (2013) 54:813–20. doi: 10.1167/iovs.11-8379, PMID: 23307967 PMC3562120

[13] Kellar KL Iannone MA. Multiplexed microsphere-based flow cytometric assays. Exp Hematol. (2002) 30:1227–37. doi: 10.1016/S0301-472X(02)00922-0, PMID: 12423675

[14] Huang XJ Liu DH Liu KY Xu LP Chen H Han W. Haploidentical hematopoietic stem cell transplantation without in vitro T-cell depletion for the treatment of hematological malignancies. Bone Marrow Transplant. (2006) 38:291–7. doi: 10.1038/sj.bmt.1705445, PMID: 16883312

[15] Oliveira-Soto L Efron N. Morphology of corneal nerves using confocal microscopy. Cornea. (2001) 20:374–84. doi: 10.1097/00003226-200105000-00008, PMID: 11333324

[16] Goldfarb M. Functions of fibroblast growth factors in vertebrate development. Cytokine Growth Factor Rev. (1996) 7:311–25. doi: 10.1016/S1359-6101(96)00039-1, PMID: 9023055

[17] Gasser E Sancar G Downes M Evans RM. Metabolic messengers: fibroblast growth factor 1. Nat Metab. (2022) 4:663–71. doi: 10.1038/s42255-022-00580-2, PMID: 35681108 PMC9624216

[18] Reneker LW Wang L Irlmeier RT Huang AJW. Fibroblast growth factor receptor 2 (FGFR2) is required for Meibomian gland homeostasis in the adult mouse. Invest Ophthalmol Vis Sci. (2017) 58:2638–46. doi: 10.1167/iovs.16-21204, PMID: 28510629 PMC5444547

[19] Yang X Zhong X Huang AJ Reneker LW. Spontaneous acinar and ductal regrowth after Meibomian gland atrophy induced by deletion of FGFR2 in a mouse model. Ocul Surf. (2022) 26:300–9. doi: 10.1016/j.jtos.2021.11.005, PMID: 34798325 PMC10317283

[20] Elabd SS Abo-Elnasr SE Soliman GM Sarhaan NI Tawfik SM. Histological study of the effect of granulocyte colony-stimulating factor on experimentally induced corneal burn in adult male albino rats. Ultrastruct Pathol. (2020) 44:116–29. doi: 10.1080/01913123.2020.1713949, PMID: 32081069

[21] Chitu V Biundo F Stanley ER. Colony stimulating factors in the nervous system. Semin Immunol. (2021) 54:101511. doi: 10.1016/j.smim.2021.101511, PMID: 34743926 PMC8671346

[22] Shima C Adachi Y Minamino K Okigaki M Shi M Imai Y. Neuroprotective effects of granulocyte colony-stimulating factor on ischemia-reperfusion injury of the retina. Ophthalmic Res. (2012) 48:199–207. doi: 10.1159/000340059, PMID: 22868688

[23] Sehgal A Irvine KM Hume DA. Functions of macrophage colony-stimulating factor (CSF1) in development, homeostasis, and tissue repair. Semin Immunol. (2021) 54:101509. doi: 10.1016/j.smim.2021.101509, PMID: 34742624

[24] Rho CR Park MY Kang S. Effects of granulocyte-macrophage Colony-stimulating (GM-CSF) factor on corneal epithelial cells in corneal wound healing model. PLoS One. (2015) 10:e0138020. doi: 10.1371/journal.pone.0138020, PMID: 26376304 PMC4574106

[25] Dohlman TH Ding J Dana R Chauhan SK. T cell-derived granulocyte-macrophage Colony-stimulating factor contributes to dry eye disease pathogenesis by promoting CD11b+ myeloid cell maturation and migration. Invest Ophthalmol Vis Sci. (2017) 58:1330–6. doi: 10.1167/iovs.16-20789, PMID: 28241321 PMC5341624

[26] Ueno H Hattori T Chi HH Miyabe Y Murayama MA. Clodronate liposome treatment contributes to the nerve regeneration in corneal nerve involvement of diabetic mice. Exp Anim. (2025) 74:58–65. doi: 10.1538/expanim.24-0063, PMID: 39111878 PMC11742477

[27] Chu DT Bac ND Nguyen KH Tien N Thanh V Nga V. An update on anti-CD137 antibodies in immunotherapies for Cancer. Int J Mol Sci. (2019) 20:1822. doi: 10.3390/ijms20081822, PMID: 31013788 PMC6515339

[28] Gilbertson DG Duff ME West JW Kelly JD Sheppard PO Hofstrand PD. Platelet-derived growth factor C (PDGF-C), a novel growth factor that binds to PDGF alpha and beta receptor. J Biol Chem. (2001) 276:27406–14. doi: 10.1074/jbc.M101056200, PMID: 11297552

[29] Tang Z Arjunan P Lee C Li Y Kumar A Hou X. Survival effect of PDGF-CC rescues neurons from apoptosis in both brain and retina by regulating GSK3beta phosphorylation. J Exp Med. (2010) 207:867–80. doi: 10.1084/jem.20091704 20231377 PMC2856029

[30] Rocco ML Soligo M Manni L Aloe L. Nerve growth factor: early studies and recent clinical trials. Curr Neuropharmacol. (2018) 16:1455–65. doi: 10.2174/1570159X16666180412092859, PMID: 29651949 PMC6295934

